# Prescription Refill Adherence Before and After Patient Portal Registration in Among General Practice Patients in England Using the Clinical Practice Research Datalink: Longitudinal Observational Study

**DOI:** 10.2196/50294

**Published:** 2025-03-11

**Authors:** Abrar Alturkistani, Thomas Beaney, Geva Greenfield, Ceire E Costelloe

**Affiliations:** 1 Department of Primary Care and Public Health, Imperial College, Reynolds Building, London, W6 8RP, United Kingdom, 44 0207589; 2 Division of Clinical Studies, Institute of Cancer Research, London, United Kingdom

**Keywords:** patient portals, personal health records, general practice, delivery of health care, medication ordering, health care, medication adherence, clinical practice research, patient portal, England, longitudinal analysis, patient health, medication, cardiovascular disease, diabetes, chronic kidney disease

## Abstract

**Background:**

Patient portal use has been associated with improved patient health and improved adherence to medications, including statins. However, there is limited research on the association between patient portal registration and outcomes such as statin prescription refill adherence in the context of the National Health Service of England, where patient portals have been widely available since 2015.

**Objective:**

We aimed to explore statin prescription refill adherence among general practice patients in England.

**Methods:**

This study was approved by the Clinical Practice Research Datalink Independent Scientific Advisory Committee (ID: 21_000411). We used patient-level general practice data from the Clinical Practice Research Datalink in England. The data included patients with cardiovascular disease, diabetes, and chronic kidney disease, who were registered on the patient portal. The primary aim was to investigate whether statin prescription refill adherence, defined as ≥80% adherence based on the medication possession ratio, improved after patient portal registration. We used a multilevel logistic regression model to compare aggregate adherence 12 months before and 12 months after patient portal registration.

**Results:**

We included 44,141 patients in the study. The analysis revealed a 16% reduction in the odds of prescription refill adherence 12 months after patient portal registration (odds ratio [OR]: 0.84, 95% CI 0.81-0.86) compared to 12 months before registration in the fully adjusted model for patient- and practice-level variables.

**Conclusions:**

This study evaluated prescription refill adherence after patient portal registration. Registering to the portal does not fully explain the mechanisms underlying the relationship between patient portal use and health-related outcomes such as medication adherence. Although a small reduction in prescription refill adherence was observed, this reduction disappeared when the follow up time was reduced to 6 months. Given the limitations of the study, reduction in prescription refill adherence cannot be entirely attributable to patient portal registration. However, there may be potential confounding factors influencing this association which should be explored through future research.

## Introduction

Patient portals are online websites or applications that are tethered to the health care system’s electronic health record and typically offer services such as online appointment booking and repeat prescription ordering. It is suggested that patient portal use may improve patients’ health through factors such as improving patients’ efficiency, information access, and continuity of care. The mechanisms involved in patient portal use can facilitate communication and could help improve patients’ health. For example, in a large integrated health care system in the United States, patients who found the use of patient portals to be more convenient than in-person care were more likely to report improved health as a result of using patient portals [1]. Patient portals provide access to medical record data on information such as diagnoses and test results and may include patient education information on managing specific health conditions or general health advice. Furthermore, information access could also include collation of patients’ information such as medications and consultations in one place, allowing patients to better manage their care [1], engage in shared decision-making, and understand their treatments [2]. Additionally, simple functionalities such as appointment booking and access to appointment records may help patients avoid missing appointments and enabling continuity of care [3].

It is hypothesised that patient portal use could improve medication adherence. A study among patients with diabetes reported that time without medication was reduced for those who exclusively used the refill function of the portal compared to those who did not use it [4]. Nonadherence to statins reduced by 6% (95% CI 4%‐7%) among patients with diabetes exclusively using the refill function compared to nonusers of the portal [5]. However, this relationship has not been studied within the National Health Service (NHS) in England, where all patient portals have been required to be offered in all General Practice (GP) practices since 2015 [6]. Studies reporting the relationship between patient portal use and medication adherence usually focus on specific functionalities of the portal. For example, studies may report the relationship between access to medical records, ordering medication through the patient portal (mainly repeat prescriptions), receiving reminder text messages, or booking appointments online. Studies also reported outcomes of patient portal use when coupled with another intervention such as telemedicine (technologies that enable remote medical consultations) or other digital interventions such as health monitoring devices or educational materials [7,8]. For example, a study found that digital technologies used for telemonitoring (which can be a feature of patient portals) that included monitoring patient health metrics and helping patients remember the time and type of medication improved medication adherence among patients with hypertension [7]. Additionally, telemonitoring may offer more accurate information on blood pressure values among patients with hypertension, which has been linked to improve therapeutic adherence to prescribed medications [7]. The digital technologies referenced in this study included apps, wearable decides, telemedicine, and digital monitoring tools (which include recording and reporting of health metrics to the health care system), and digital rehabilitation [7]. While the intervention considered in this study only includes access to medical records (Multimedia Appendix 1), descriptions of patient portals in the NHS (also known as online services) may also include features such as telemedicine, access to patient education materials, ability to book appointments, and ordering repeat prescriptions [6,9-11].

Statins are prescribed to patients both for primary prevention or secondary prevention of cardiovascular events; they are typically taken once a day and mostly continued for life, once prescribed to the patient [12]. Statin adherence could lower the risk of mortality [13]. Conversely, low adherence to statins increases the risk of death in the second year after a myocardial infraction and is also associated with increased risk of cardiovascular and cancer mortality [14-16]. A systematic review on factors associated with statin adherence for primary prevention of cardiovascular disease (CVD), included age, gender, or sex, diabetes status, hypertension, socioeconomic status, level of education, geographical region, race, and marital status [17]. This review also added that factors such as presence of dyslipidemia (high cholesterol), smoking status, work status, and comorbidity are either not associated or have mixed associations with statin adherence [17]. In this study we aimed to determine whether adherence to prescription refill statins improved after registering to patient portals among general practice patients using the Clinical Practice Research Datalink (CPRD), focusing on patients with adverse cardiovascular events, chronic kidney disease (CKD), CVD, and diabetes. This article is based on Chapter 4 of the author’s PhD thesis [18].

## Methods

### Ethical Considerations

The study protocol was approved by CPRD’s Independent Scientific Advisory Committee (ID: 21_000411). In the context of CPRD data usage, informed consent is not required for the anonymized data provided to CPRD from participating GP practices. The data is anonymized to the extent that individual patients cannot be identified. Participants have the option to opt out of having their health records shared for research purposes [19]. The original study approval covers secondary analyses, eliminating the need for additional consent from participants. No compensation was provided to participants in this study. Approved researchers could gain access to the data after submitting a study protocol.

### Study Design

This was an observational study using longitudinal data on prescribed medication (statins) extracted from electronic health record data to compare patient-level aggregate adherence 12 months before and 12 months after patient portal registration, Adherence was measured using the medication possession ratio (MPR) with a threshold of 80% as an indicator of adherence. The data source, CPRD Aurum, comprises electronic health record data from GPs in England, where GPs are the gatekeepers of the health care system, and almost all of the population is registered with a GP [20]. The study period varied by patient and ranged from January 1, 2014, to August 15, 2021.

### Study Population

To determine our population, we first included all patients in CPRD Aurum who had a patient portal registration code (available in Multimedia Appendix 1) recorded on or after January 1, 2015, when online services were required to be offered by all GPs in England [6]. Eligible patients had a diagnosis of at least one of the following conditions: adverse cardiovascular event, CKD, CVD, or diabetes, using codes in the study by Davidson et al [21]. These patient groups were chosen because they may be prescribed statins according to National Institute for Health and Care Excellence (NICE) guidelines for CVD prevention [22]. Although not all patients with diabetes will be prescribed statins, we included them due to the interest in the literature on the association between patient portal use and diabetes-related outcomes, including statin adherence [23].

### Variables

#### Outcome Variable

The statin prescription refill adherence was determined through several steps. First, we extracted the codes of statin prescriptions using the codes provided in the study by Davidson et al [21], and aggregated the duration variable (defined as duration of treatment in days)for the period of one year before and one year after patient portal registration [24]. We assumed that if a patient duration was recorded as equal to zero, it was either entered by error or that it did not represent an actual statin prescription, as medication cannot be prescribed for zero days; therefore these observations were removed. Next, we calculated the MPR, which is a measure created by dividing the number of days of supply of the prescribed medication for all prescriptions within a time interval by the time interval [25]. In our study, we divided the aggregated duration by 365 (ie, 1 yr-period). The MPR was calculated twice for each patient for each time-period—one year before and one year after patient portal registration. We assumed that patients who did not have any record of statin prescriptions either before or after patient portal registration, to have an adherence of zero for that period. The MPR was then categorized into a binary variable of prescription refill adherence, with an 80% threshold by considering all values of ≥80% as adherent, and those <80% as nonadherent. The 80% cut off point was chosen to create easy-to-interpret binary variable for adherence following the same measure used in a previous CPRD study [26], as recommended research that determined 80% MPR to be a reasonable cut off point for categorizing the patient as adherent or nonadherent [27]. However, we have considered other cut off points, including 50% and 65% to compare the differences between these measures. The same logic was applied for adherence values of 65% and 50%. The various adherence values only indicate a change in the level of MPR that is considered adherent. Patients were considered adherent at the 80% threshold if they had an MPR ≥80%, at 65% if they had an MPR ≥65%, and at 50% if they had an MPR ≥50%. Patients having an MPR of ≥80% were considered adherent at all lower thresholds.

#### Predictors

The description of other variables included in the study including the predictor variable (ie, patient portal registration status) are described in Table 1. While multiple factors are associated with statin adherence, the research team decided to use the same variables included in our previous studies on at factors associated with patient portal use in NHS GP practice settings [28]. We also included all of the factors that are essential to the model such as gender, age group, ethnicity, and level of deprivation, which are associated with both patient portal use and medication refill adherence [17,28]. Hearing loss was added as a predictor because it can be associated with patient portal use and has been included in other studies on patient portal use in NHS GP settings [28-30].

**Table 1.** Description of study variables, their categories, and sources. Adapted from [18], under CC BY-NC license [31].

Variables	Description and categories
Gender	Gender is provided by Clinical Practice Research Datalink (CPRD) and consists of 3 categories including male, female, and indeterminate [24]. However, there were no patients with the indeterminate gender category in this cohort.
Patient portal registration status	We identified 6 codes that indicated registration or access to patients’ online medical record and used them as a proxy for patient portal registration in CPRD to search patients’ observation files for identifying the codes that indicate patient portal registration (Multimedia Appendix 1). We used registration to patient portals as a proxy for patient portal use due to lack of data in CPRD on the actual usage of patient portals.
Date of patient portal registration	The date of patient portal registration was determined as the observation date of the patient portal registration code. As patients only needed to register to the portal once, most patients only had one observation for patient portal registration. For those that had more than one code and therefore more than one observation date, we maintained the latest observation date as the patient portal registration date.
Age at registration age groups	CPRD provided only birth-year record of patients over 16 years. To calculate age, we assumed all patients were born on July 1 and calculated their age in reference to their patient portal registration date. We categorized age into 6 age groups: 16-44, 45-54, 55-64, 65-74, 75-84, and >85 years.
Self-reported ethnicity	Self-reported ethnicity was extracted from the observation files of patients using code list for ethnicity provided in the study by Davidson et al [32]. Ethnicity was categorized into 5 Office for National Statistics groups: White, Black, Mixed, South Asian, and other. Patients who did not have a code for ethnicity were placed in the 6th category “unknown.” Patients who had more than one code for ethnicity were categorized into the ethnicity that had the majority of codes. If patients had multiple ethnicities with an equal number of codes for each ethnicity category, we assigned them the most recent ethnicity category as their ethnicity.
Index of multiple deprivation (IMD) quintiles	IMD 2019 was calculated using patients’ postcodes and was provided by CPRD as linkage data on request.
Hearing loss	Hearing loss codes were extracted from the study by Head et al [33] using patients’ observation files, and a binary variable was created to indicate whether the patient had hearing loss.
General practice rurality	2011 rural-urban classification at lower layer super output area level.

### Statistical Methods

All analyses were performed using RStudio software (version 2023.06.0+421; R Foundation for Statistical Computing). We compared statin prescription refill adherence (measured by MPR ≥80%) 12 months before and 12 months after patient portal registration using multilevel logistic regression. We assumed that once patients were prescribed statins they would be for long-term use, indicating that they should have ordered statins consistently over the 12-month period before and after patient portal registration. The multilevel logistic regression was performed in three major steps:

1. First, we created a null model with only the outcome variable and added GP practice as the grouping variable. This step helped observe if clustering by GP was present. We checked the interclass correlation coefficient (ICC), which ranged from 0‐1. A value of 0 ICC indicates no clustering at the group level and a value of 1 indicates perfect clustering at the group level.
2. At step 2, we added patient-level characteristics, as multilevel modeling requires separate steps for variables from two different levels (in this case, patients at level 1 and GP practices at level 2) [34]. Therefore, step we added all the predictor variables that were at the patient-level, including gender (male, female), age group (16-44, 45-54, 55-64, 65-74, 75-84, >85 years), self-reported ethnicity (White, Black, mixed, South Asian, and other), Index of Multiple Deprivation (IMD) quintiles (1-5), and hearing loss (yes, no).
3. At step three, we added rurality of the GP practice as the GP practice-level variable.

### Sensitivity Analysis

We reduced the follow up time to 6 months instead of 12 months before and after patient portal registration to examine potential differences in association. Additionally, we performed a sensitivity analysis including all patients that were initially excluded from the study due to missing IMD measure data.

## Results

### Study Size

**Figure 1.**
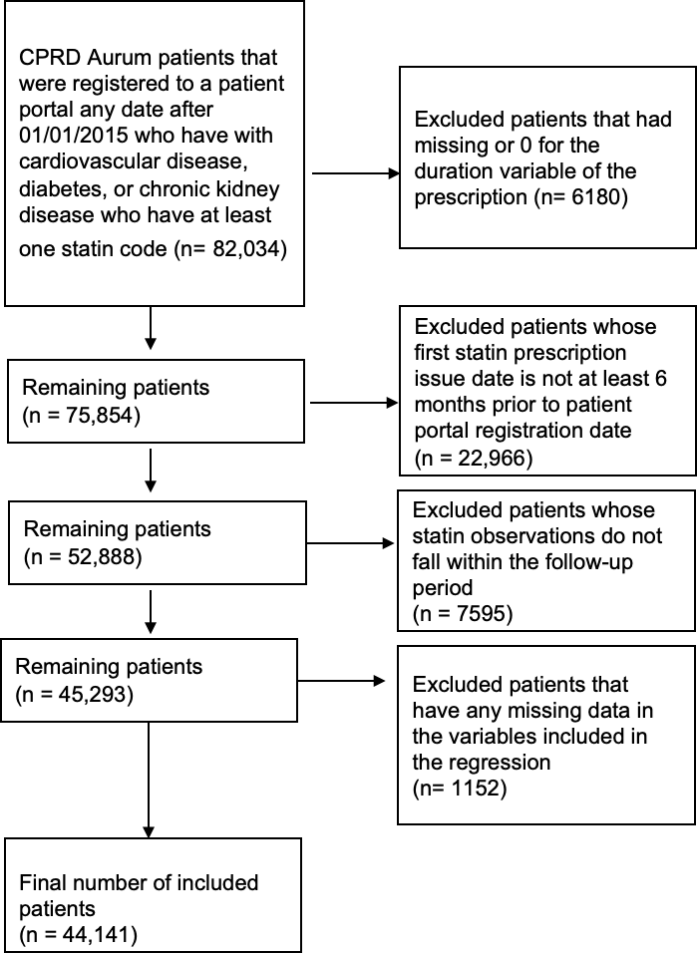
Flow chart of the study population and excluded patients.

Patients who had missing duration or 0-duration prescriptions were excluded (n=6180) (Figure 1). We excluded all patients whose first statin prescription was recorded at least 12 months before patient portal registration date (n=22,966) (Figure 1) to ensure that patients had at least 12 months of follow-up before patient portal registration. All patients whose statin observations did not fall within the required follow-up period (12 months before and 12 months after patient portal registration date) were excluded (n=7595) (Figure 1). Finally, we only included patients with complete data in the analysis, and excluded all patients that had missing IMD quintile data (n=44,141) (Figure 1). There were no missing data in any other variable except ethnicity, which was included as a separate category in the analysis.

Figure 1 is reused from PhD Thesis by Alturkistani [18].

### Summary Statistics

A total of 44,141 patients were included in the study. The majority (n=13,812) of the patients were men aged 65‐74 years at the time of patient portal registration and had a prescription refill adherence of 80% or higher (n=27,208) (Table 2).

**Table 2.** Summary statistics of the study population. Reused from Alturkistani [18] under CC BY-NC license [31].

Characteristics	Patients (N=44,141), n (%)
Gender	
Men	26,620 (60.3)
Women	17,521 (39.7)
Age groups (years)	
16-44	1787 (4)
45-54	5550 (12.6)
55-64	11,308 (25.6)
65-74	13,812 (31.3)
75-84	8755 (19.8)
>85	2929 (6.6)
Prescription refill adherence (medication possession ratio ≥80%)	
No	16,933 (38.4)
Yes	27,208 (61.6)
Ethnicity	
White	32,547 (73.7)
South Asian	4664 (10.6)
Black	1601 (3.6)
Mixed	340 (0.8)
Other	436 (1)
Unknown	4553 (10.3)
IMD^a^ quintile (1-5)^b^	
1	8041 (18.2)
2	9507 (21.5)
3	8697 (19.7)
4	9288 (21)
5	8608 (19.5)
Location of GP^c^	
Urban	38,054 (86.2)
Rural	6087 (13.8)
Hearing loss condition	
No	25,198 (57.1)
Yes	18,943 (42.9)

a IMD: Index of Multiple Deprivation.

b Quintiles: 1 (most deprived) to 5 (least deprived).

c GP: General practice.

### Adherence Summary Statistics

The mean MPR 12 months before patient portal registration was 87% and ranged from 0%-183%, while the mean MPR 12 months after patient portal registration was 81%, ranging from 0%-164%. The median MPR was 92%, both before and after patient portal registration.

### Association Between Patient Portal Registration and Statin Prescription Refill Adherence

Table 2 presents the results of the fully adjusted, multilevel logistic regression for the outcome variable (statin prescription refill adherence of ≥50%, ≥65%, and ≥80%). The odds ratio (OR) for statin prescription refill adherence of ≥80% was 0.84 (95% CI 0.81-0.86), indicating a 16% reduction in the odds of statin prescription refill adherence at this threshold after patient portal registration, compared to 12 months before registration (Table 3). The OR for adherence at 50% and 65% thresholds also indicated a reduction in adherence after patient portal registration, indicating that even patients with lower baseline adherence were less likely to remain adherent after patient portal registration (Table 3). The ICC varied for different levels of adherence but were generally 15% or lower, indicating the proportion of adherence variance that can be attributable to practice-level factors. For example, an ICC of 15% can be interpreted as 15% of the total variance in adherence by patients can be explained by differences at the GP practice level.

**Table 3.** Results of the fully-adjusted multilevel logistic regression model of patient portal registration status on statin prescription refill adherence of ≥50%, ≥65%, and ≥80% (level 1, N=88,282 observations of 44,141 patients; level 2, n=1190 general practices) 12 months before and 12 months after patient portal registration. Reused from [18] under CC BY-NC license [31].

Predictors	Adherence ≥50%		Adherence ≥65%		Adherence ≥80%	
	OR (95% CI)	*P* value	OR (95% CI)	*P* value	OR (95% CI)	*P* value
Patient portal registration status (after registration)	0.79 (0.77-0.82)	<.001	0.83 (0.81- 0.86)	<.001	0.84 (0.81-0.86)	<.001
Gender- (Ref: Male)						
Women	0.96 (0.93-0.99)	0.01	0.98 (0.95-1.01)	0.30	1.00 (0.97-1.03)	0.85
Age group, years (Ref: 65‐74)						
16‐44	0.46 (0.42-0.49)	<.001	0.43 (0.39-0.46)	<.001	0.40 (0.37-0.43)	<.001
45‐54	0.67 (0.64-0.71)	<.001	0.65 (0.62-0.68)	<.001	0.60 (0.58-0.63)	<.001
55‐64	0.84 (0.80-0.87)	<.001	0.84 (0.81-0.88)	<.001	0.78 (0.75-0.81)	<.001
75‐84	1.08 (1.03-1.13)	0.002	1.08 (1.04-1.13)	<.001	1.08 (1.04-1.13)	<.001
>85	0.82 (0.77-0.88)	<.001	0.89 (0.83-0.95)	<.001	0.95 (0.90-1.02)	0.14
Ethnicity (Ref: White)						
Asian	0.88 (0.83-0.94)	<.001	0.90 (0.84-0.96)	<.001	0.89 (0.84-0.94)	<.001
Black	0.62 (0.56-0.68)	<.001	0.59 (0.54-0.65)	<.001	0.60 (0.55-0.65)	<.001
Mixed	0.77 (0.65-0.92)	0.003	0.72 (0.61-0.85)	<.001	0.72 (0.61-0.84)	<.001
Other	0.81 (0.70-0.95)	0.01	0.85 (0.74-0.99)	0.04	0.82 (0.71-0.95)	0.01
Unknown	1.08 (1.02-1.15)	0.01	1.10 (1.04-1.17)	<.001	1.09 (1.04-1.15)	<.001
IMD^a^ quintile^b^ (Ref: 5)						
1	0.93 (0.87-0.99)	0.03	0.93 (0.87-0.99)	0.02	0.95 (0.89-1.00)	0.07
2	0.90 (0.85-0.96)	0.001	0.90 (0.85-0.95)	0.00	0.91 (0.86-0.96)	0.001
3	0.91 (0.85-0.96)	0.001	0.91 (0.86-0.96)	0.001	0.92 (0.87-0.97)	0.002
4	0.97 (0.91-1.02)	0.23	0.94 (0.90-1.00)	0.03	0.94 (0.89-0.99)	0.01
Hearing loss (Ref: No)						
Yes	1.01 (0.98-1.05)	0.48	1.03 (0.99-1.06)	0.10	1.00 (0.97-1.03)	0.78
General practice location(Ref: Urban)						
Rural	1.02 (0.88-1.17)	0.82	1.04 (0.88-1.22)	0.65	1.00 (0.86-1.16)	0.99
Interclass correlation coefficient	0.10		0.15		0.13	

a IMD: Index of Multiple Deprivation.

b Quintiles 1 (most deprived) to 5 (least deprived).

### Results of the Sensitivity Analysis

When the time of follow up was reduced to 6 months, the OR for statin prescription refill adherence of ≥80% was 1.01 (95% CI 0.98-1.04) in the fully-adjusted model, indicating no difference in statin prescription refill adherence 6 months after patient portal registration compared to the 6 months before patient portal registration (Multimedia Appendix 2).

The OR differed by only 0.01‐0.02 of sensitivity analyses, which included patients who had missing IMD quintiles data (results not shown).

## Discussion

### Main Findings of the Study

After registering to the patient portal, patients had lower odds of achieving adherence of 50%, 65%, and 80% thresholds. However, this relationship was attenuated when the follow-up time was reduced to 6 months before and after patient portal registration and indicated no change in adherence after registration. This study found a reduction in prescription refill adherence after patient portal registration, which may be due to several reasons. Patients in this study were only registered to the portal once, and it is likely that may have enrolled at a time of high health care utilization. This could lead to increased medication requests prior to patient portal registration, compared to after registration. Additionally, patients may have overall reduced adherence to statins after using it for some time due to factors such as side effects, continuity of care, disease severity, or socioeconomic factors, which could all affect adherence to ordering prescriptions [26,27].

### Comparison to Prior Work

Previous studies revealed that factors such as experiencing side effects, CVD severity, continuity of care and income, or other indicators of deprivation may influence adherence to statins [26,27]. Other factors that could be associated with statin adherence but were not examined in this study include the type of statin, intensity of the dose, timing of prescribing statins (eg, before or after puberty) [17]. Given that statin adherence reduced when patients were followed up for one year but not over 6 months, it is possible that statin adherence naturally reduced over time. Since the codes used for patient portal registration in this study indicated to access medical records, we could not explore potential mechanisms involved between accessing medical records and medication adherence. We acknowledge this as a limitation of the study, as registration to the patient portal does not necessarily mean continued use of the patient portal, discussed further in the limitations and strengths subsection [35].

Among features of patient portals, the ability to order prescriptions through the portal is most strongly associated with medication adherence. For example, one study reported that when medication is ordered through a patient portal, it can have less errors and more clear instructions (compared to hand-written prescriptions), which in turn improves adherence to treatment [36]. Having a higher supply of statin (eg, a supply of 60 d vs 30 d) was associated with improved adherence, which can be achieved by using a patient portal (by providing instant access to ordering medication) [17].

### Limitations and Strengths of the Study

This study is relevant in the wake of increased popularity of using patient portals and other remote or digital health care services, particularly during and post-COVID-19 pandemic, both in England and other countries. Although widely offered within the NHS, this technology is rarely studied. We were unable to study active or continuous patient portal use, as this information was not recorded in CPRD, despite it being a rich source of data for clinical patient records. This study highlights the need to improve recording of the use of technologies to enable research in this field. To the authors’ knowledge, this is the first study using CPRD data to examine patient portal use. Although the study was only able to capture registration to patient portals, the records of registration were obtained from an objective source (ie, electronic health records), which is a reliable method.

A strength of this study was that it controlled for clustering of patients within GP practices by using a multilevel model and including GP practices as a fixed effect, using a model which controls for the hierarchy of the data [37]. This could be attributed to patients from the same practice being more similar to each other, patient portals varying in functionalities, and knowledge and encouragement of their staff including GPs in using the patient portal [9].

One limitation in our study was that we could not measure or report the differences in patient portal functionalities that were included in the study, as our study included more than 1000 GP practices. Despite this, by including a large number of practices, we were able to analyze a more representative sample. We attempted to account for the differences between practices by running a multilevel model, recognizing that patients from the same GP practice may have more similar outcomes due to various factors including the type of patient portal offered in the practice and type of education and training available to both staff and patients [9].

While registration to the patient portal is the first step needed to benefit from its functionalities, studies show that without continuous or active use, patients cannot benefit from the technology. Registering to the portal does not guarantee continuous use of the portal, [38] which is essential to observe benefits associated with patient portal use [39]. A major limitation of this study was only using codes that indicated registration for accessing medical record as a proxy for patient portal use. This limited our study in two ways. First, access to medical records may have a minimal impact on prescription refill adherence. Second, patient portals offered through the NHS GPs, typically offer various functionalities beyond access to medical records, including online appointment booking and repeat prescription ordering [40]. However, as described in Multimedia Appendix 1, our search for codes that indicate patient portal use in CPRD Aurum only identified codes that indicate access to the medical records. This presented a challenge as patients using other portal functionalities, such as online appointment booking and prescription ordering, were not reflected in their electronic health records. Consequently, we could not determine if patients without these specific codes (codes in Multimedia Appendix 1) were indeed nonusers of patient portals and therefore, suitable as controls for our study. While we recognize that this limitation reduces the robustness of the results, it was the most suitable approach to examine this relationship that remains unexplored within the NHS, despite a wide availability of patient portals for nearly a decade. Further information about the use of patient portals such as prescription refill requests or appointment bookings made through the portal could better inform the relationship between patient portal use and medication adherence. Additionally, our study population included patients with chronic diseases including CVD, CKD, and diabetes, who may be less likely to use patient portals and more likely to enroll or register to patient portals [41]. According to a study conducted in the United States, patients with certain circulatory diseases such as heart failure, myocardial infraction, peripheral vascular disease, and those with diabetes and renal disease were less likely to use the patient portal of the health care system [42]. Given that patients included in this study included those with CVD, CKD, and diabetes, it is possible that although they were registered to the patient portal, these patients may not have been active users. Therefore, we cannot conclude whether the patient experienced reduced adherence to refilling their prescriptions due to the patient portal use based solely on the findings from this study.

### Conclusion

In this study, patients had a slightly reduced adherence one year after patient portal registration, although adherence did not change after a 6 months follow up. This could be attributed to other mechanisms, including those mentioned above, that could influence medication adherence and were not included in this study. Registration to the portal alone does not influence health-related behaviors such as medication adherence. Future studies on recording of patient portal use in electronic health records such as log files of functionality use in patient portals (eg, appointment booking, messaging health care providers, or requesting medications) could enable studying health related outcomes and explain mechanisms involved between patient portal use and health outcomes.

## Supplementary material

10.2196/50294 Multimedia Appendix 1 Patient portal registration codes used to extract patient records from CPRD Aurum.

10.2196/50294 Multimedia Appendix 2 Results of the fully adjusted multilevel logistic regression model of patient portal registration status on statin ordering adherence (medication possession ratio) of 50% or more, 65% or more, and 80% or more (level 1, N= 89,064 observations of 44,532 patients; level 2, N=1188 general practices) with 6 months before and 6 months after patient portal registration.

10.2196/50294 Checklist 1 The reporting of studies conducted using observational routinely collected health data checklist.

## Abbreviations:

CKD: chronic kidney disease
CPRD: Clinical Practice Research Datalink
CVD: cardiovascular disease
GP: general practice
IMD: index of multiple deprivation
MPR: medication possession ratio
NHS: National Health Service
NICE: The National Institute for Health and Care Excellence
OR: odds ratio
