# Supplementary material for: Prescription Refill Adherence Before and After Patient Portal Registration in Among General Practice Patients in England Using the Clinical Practice Research Datalink: Longitudinal Observational Study
Source: JMIR Med Inform. 2025 Mar 11;13:e50294. doi: 10.2196/50294 (PMC11918981; doi:10.2196/50294)
Supplement: Multimedia Appendix 1 [file medinform-v13-e50294-s001.docx]

Multimedia Appendix 2: patient portal registration codes used to extract patient records from CPRD Aurum

The information shared in this document is also shared in another paper [1] using the same main data source (CPRD Aurum of patients with patient portal registration code)

Prior to performing the study we have searched the CPRD bibliography to identify any publications that studies patient portals in CPRD to identify code lists of patient portal registration or use. No studies were found at the time of performing the study that provided code lists for patient portal registration. Therefore, as recommended by CPRD, we have performed manual searches of the CPRD Aurum medical dictionary code browser for words such as: patient portal, Portal, Internet, Repeat, Remote, App, Appointment, service, *online*, *digital*, *electronic*, *application*, *record*. The final codes that were used in this study are in the table below:

| **MedCodeId** | **Term** | **SnomedCTConceptId** | **SnomedCTDescriptionId** |
| --- | --- | --- | --- |
| 2405101000000115 | Registered for online access to local practice | 939511000000101 | 2405101000000115 |
| 1849591000006114 | Patient has online access to primary care medical record | 1849591000006105 | 1849591000006114 |
| 1573261000006118 | Patient local record access enabled | 1573261000006102 | 1573261000006118 |
| 7805631000006116 | Registration for online access to local general practice service | 720582003 | 3321317011 |
| 1950401000006118 | Patient offered online access to primary care medical record | 1950401000006102 | 1950401000006118 |
| 1573241000006117 | Patient remote record access enabled | 1573241000006101 | 1573241000006117 |

[1] Alturkistani A, Beaney T, Greenfield G, Costelloe CE, Patient portal registration and healthcare utilisation in General Practices in England (In press). BJGP Open.
