# Supplementary material for: Prescription Refill Adherence Before and After Patient Portal Registration in Among General Practice Patients in England Using the Clinical Practice Research Datalink: Longitudinal Observational Study
Source: JMIR Med Inform. 2025 Mar 11;13:e50294. doi: 10.2196/50294 (PMC11918981; doi:10.2196/50294)
Supplement: Multimedia Appendix 2 [file medinform-v13-e50294-s002.docx]

Multimedia Appendix 4:

Results of the fully-adjusted multilevel logistic regression model of patient portal registration status on statin ordering adherence (medication possession ratio) of 50% or more, 65% or more, and 80% or more (level 1, N= 89,064 observations of 44,532 patients; level 2, N=1188 general practices) with 6 months before and 6 months after patient portal registration

|  | *adherence≥50%* | | *adherence≥65%* | | *adherence≥80%* | |
| --- | --- | --- | --- | --- | --- | --- |
| *Predictors* | *Odds Ratios* | *CI* | *Odds Ratios* | *CI* | *Odds Ratios* | *CI* |
| patient portal registration status- after registration | 0.99 | (0.96, 1.03) | 0.99 | (0.96, 1.02) | 1.01 | (0.98, 1.04) |
| Gender-Female (REF= Male) | 1.01 | (0.98, 1.05) | 1.01 | (0.98, 1.04) | 1.02 | (0.99, 1.05) |
| Age group (REF: 65-74) |  |  |  |  |  |  |
| 16-44 | 0.49*** | (0.45, 0.53) | 0.47*** | (0.43, 0.50) | 0.45*** | (0.42, 0.49) |
| 45-54 | 0.67*** | (0.63, 0.70) | 0.64*** | (0.61, 0.67) | 0.62*** | (0.59, 0.65) |
| 55-64 | 0.84*** | (0.80, 0.88) | 0.82*** | (0.79, 0.86) | 0.81*** | (0.78, 0.84) |
| 75-84 | 1.14*** | (1.08, 1.19) | 1.13*** | (1.08, 1.18) | 1.13*** | (1.09, 1.18) |
| 85+ | 1.01 | (0.94, 1.09) | 1.11** | (1.04, 1.19) | 1.08* | (1.01, 1.15) |
| Ethnicity (REF: White) |  |  |  |  |  |  |
| Asian | 0.88*** | (0.82, 0.94) | 0.88*** | (0.82, 0.93) | 0.89*** | (0.84, 0.95) |
| Black | 0.65*** | (0.59, 0.71) | 0.60*** | (0.55, 0.66) | 0.60*** | (0.55, 0.66) |
| Mixed | 0.67*** | (0.57, 0.80) | 0.65*** | (0.55, 0.76) | 0.72*** | (0.61, 0.84) |
| Other | 0.70*** | (0.60, 0.82) | 0.71*** | (0.61, 0.82) | 0.68*** | (0.59, 0.78) |
| Unknown | 1.12*** | (1.05, 1.19) | 1.13*** | (1.07, 1.19) | 1.11*** | (1.06, 1.17) |
| Deprivation quintile (REF: 5- Least deprived) |  |  |  |  |  |  |
| 1- Most deprived | 0.92* | (0.86, 0.99) | 0.91** | (0.85, 0.97) | 0.95 | (0.90, 1.01) |
| 2 | 0.87*** | (0.82, 0.92) | 0.87*** | (0.82, 0.92) | 0.91*** | (0.87, 0.96) |
| 3 | 0.91** | (0.85, 0.97) | 0.90*** | (0.85, 0.95) | 0.93** | (0.88, 0.98) |
| 4 | 0.93* | (0.88, 0.98) | 0.92** | (0.88, 0.97) | 0.92*** | (0.88, 0.97) |
| hearing loss-Yes (REF= No) | 0.99 | (0.96, 1.03) | 1.00 | (0.97, 1.03) | 0.99 | (0.96, 1.02) |
| General practice rurality-Rural (REF= Urban) | 1.12 | (0.96, 1.30) | 1.12 | (0.96, 1.32) | 0.95 | (0.82, 1.11) |
| Interclass correlation coefficient (ICC) | 0.13 | | 0.14 | | 0.13 | |

^*^: p-value<0.05, ^**^: p-value<0.01, ^***^: p-value<0.001
